# Prevalence and risk factors of *Mycoplasma genitalium* infection in patients attending a sexually transmitted infection clinic in Reunion Island: a cross-sectional study (2017–2018)

**DOI:** 10.1186/s12879-021-06193-6

**Published:** 2021-05-26

**Authors:** Roxane Begnis, Nicolas Bouscaren, Loic Raffray, Cécile Saint Pastou Terrier, Fanny Andry, Malik Boukerrou, Yatrika Koumar, Marie-Pierre Moiton, Patrick Gerardin, Antoine Bertolotti

**Affiliations:** 1 CHU Réunion, Service des Maladies Infectieuses – Dermatologie, Saint Pierre, La Réunion France; 2 Inserm CIC1410, CHU Réunion, Saint Pierre, La Réunion France; 3 CHU Réunion, Service de Médecine Interne – Dermatologie, Saint Denis, La Réunion France; 4 CHU Réunion, Service de Gynécologie et Obstétrique, Saint-Pierre, La Réunion France; 5 CHU Réunion, Service des Maladies Infectieuses, Saint-Denis, La Réunion France

**Keywords:** Sexually transmitted infection, *Mycoplasma genitalium*, Prevalence, Reunion Island

## Abstract

**Background:**

*Mycoplasma genitalium* (MG) is an emerging sexually transmitted infection (STI) for whose management remains controversial. We aimed to assess the prevalence and risk factors of MG infection in patients attending an STI clinic in Reunion Island.

**Methods:**

Between January 2017 and December 2018, all patients attending the Saint-Pierre STI clinic in Reunion Island were screened for MG, *Chlamydia trachomatis* (CT) and *Neisseria gonorrhoeae* (NG). Urogenital, pharyngeal and/or anal samples were collected based on sexual behaviour and analysed by triplex PCR. Risk factors were identified using a Poisson regression for binary outcome.

**Results:**

Among 2069 screened subjects, the overall prevalence of MG was 4.88% [95% Confidence Interval (CI) 3.98–5.93]. The prevalence of urogenital MG was 4.38%, with women being more affected than men (5.33% vs 3.22%, prevalence ratio (PR) 1.66, *p = 0.02*). The prevalence of anal MG was 3.06% and that of pharyngeal MG was 0.61%, with men being more affected in both cases. Infection with MG was independently associated with multiple partners (6–10 partners: adjusted prevalence ratio-aPR 2.55, *p < 0.048*; > 10 partners: aPR 4.33, *p < 0.004*), previous history of STI (aPR 1.89, *p = 0.026*), non-use of condoms (aPR 2.56, *p < 0.003*) and co-infection with CT (aPR 2.56, *p < 0.017*).

**Conclusion:**

Compared to other countries, the prevalence of MG is high in Reunion Island, especially in women aged under 25 years, and co-infection with CT is common. Routine MG screening and treatment should be performed in at-risk women and co-infection with MG should be considered when deciding on treatment for CT, particularly in regions where azithromycin is still in use.

**Supplementary Information:**

The online version contains supplementary material available at 10.1186/s12879-021-06193-6.

## Background

The prevalence of sexually transmitted infections (STIs) has been on the rise in France in the last decades [1]. In particular, *Mycoplasma genitalium* (MG) is now recognised as an emerging STI responsible for non-gonococcal urethritis in men and for vaginitis, cervicitis and pelvic inflammatory disease in women [2]. It is also associated with infertility disorders and pregnancy complications, including spontaneous abortion and preterm birth.

In Europe, the prevalence of MG infection ranges from 1 to 4% in men and from 1 to 6.4% in women [3]. In France, a 2015 study found a prevalence of 3.4% in at-risk patients attending 16 STI clinics, with a specific prevalence of 0.8% in pregnant women [4] In spite of such figures, French STI clinics do not perform routine MG screening. Indeed, screening and treatment for MG have been the subject of controversy given the growth of macrolide resistance in recent years [5, 6]. Insufficient screening is nevertheless a source of concern, especially since MG infection is asymptomatic in 40 to 75% of women and in 30% of men [7]. As regards the French overseas department of Reunion Island, no data on MG infection are available at the moment.

The aim of this study was to determine the prevalence and risk factors of MG infection in patients attending an STI clinic in Reunion Island with a view to updating current guidelines and improving public health policy.

## Study methods

### Study design and population

This cross-sectional study was conducted between January 2017 and December 2018 at the Saint-Pierre STI clinic in southern Reunion Island. All patients were routinely screened for MG, *Chlamydia trachomatis* (CT) and *Neisseria gonorrhoeae* (NG). Urogenital, pharyngeal and anal samples (swap UTM™ Copan Italy) were collected based on sexual behaviour and analysed by triplex PCR (FTD Urethritis Basic Kit, Fast Track Diagnostics, Luxembourg). Data were collected by doctors using a national standardized anonymous questionnaire part of medical records.

### Statistical analysis

Categorical variables were expressed as numbers and percentages and were compared using a chi-square test or Fisher’s exact test, as appropriate. Prevalence was expressed with a confidence interval of 95% [95% Confidence Interval (CI)]. To compare prevalence between groups and to identify risk factors for MG, crude and adjusted prevalence ratios (PRs) and their 95%CI were assessed using a Poisson regression model for binary outcome. A two-tailed *p*-value of < 0.05 was considered statistically significant. All analyses were performed using STATA V13.1®.

### Ethic statement

Oral informed consent was obtained from all participants and written informed consent was obtained from a parent or guardian for participants under 18 years old. The ethical character of oral consent alone for this study on totally anonymous collected data was approved by the Ethic Committee for research of the CHU Réunion. In accordance with French regulations, this study did not require a *Comité de Protection des Personnes* (article R1121–1, decree n°2017–884 of 9 May 2018 - art.2), but was registered with the National Institute of Health Data under the number MR 0614041119. This study was conducted according to the Declaration of Helsinki and the reference methodology MR-004 of the National Commission for Information Technology and Liberties (CNIL). In the absence of overt opposition, the French regulation allows the re-use of raw data and biological samples for care, prevention, or research purposes, with the exception of genetic samples, as specified in the welcome booklet of our teaching hospital.

## Results

### Characteristics of the studied population (Additional file 1)

Of the 2467 patients who visited the Saint-Pierre STI clinic between January 2017 and December 2018 (55.4% of whom were women and 44.6% were men), 2069 were screened for MG, CT and NG using multiplex PCR, while the others who were tested only for HIV HBV, HCV and/or syphilis. Urogenital samples were collected from 1987 patients (57.3% of samples from women and 42.7% from men), pharyngeal samples from 1136 patients (17.9% of samples from men and 82.1% from women) and anal samples from 327 patients (41.9% of samples from women and 58.1% from men). The age distribution of cases was 9.2% in patients aged under 18 years, 32.2% in patients aged 18–25 years, 20.1% in patients aged 25–30 years and 38.5% in patients aged over 30 years. A previous history of STI was reported in 35.6% of patients. Urethritis or cervicitis was observed in 8 patients, 2 of whom were co-infected with CT and one was co-infected with NG. No patient receiving HIV pre-exposure prophylaxis was infected with MG.

### Prevalence of MG by sex, age and sample type (Additional file 1)

The overall prevalence of MG was 4.88% [95%CI 3.98–5.93] (101/2969) and was higher in women than men (5.53% (65/1176) vs 4.13% (36/871); PR 1.34 [95%CI 0.89–2.01], *p = 0.16*)*.*

The prevalence of urogenital MG was 4.38% [95%CI 3.51–5.40] (87/1987) and was higher in women than men (5.33% (60/1126) vs 3.22% (27/839); PR 1.66 [95%CI 1.05–2.61], *p = 0.02*). The prevalence of anal MG was 3.06% [95%CI 1.47–5.62] (10/327), with no difference between women and men who have sex with men (MSM) (2.24% (3/134) vs 3.76% (7/186); PR 0.59 [95%CI 0.15–2.30]), *p = 0.45*). The prevalence of pharyngeal MG was 0.61% [95%CI 0.25–1.27], with no difference between women and MSM (0.54% vs 1%; PR 0.54 [95%CI 0.11–2.81], *p = 0.47)*.

The prevalence of MG increased with age in patients aged under 30 years, regardless of sample type or sex. A statistically significant difference in prevalence between age groups was found only for vaginal samples (*p = 0.03*).

### Prevalence of MG by sexual behaviour and social status (Additional file 1) and by co-infection

Patients engaging in high-risk sexual behaviour were more likely to be infected with MG. A positive linear association was found between multiple partners (2 partners: PR 2.39 [95%CI 1.03–4.70]; 3–5 partners: PR 4.49 [95%CI 2.70–7.01]; 6–10 partners: PR 6.21 [95%CI 2.98–11.42]; > 10 partners: PR 12.50 [95%CI 5.40–24.63], *p < 0.001*) and the risk of MG infection. Similarly, a previous history of STI (PR 2.38 [95%CI 1.38–4.11], *p = 0.002*) and precarity (PR 2.35 [95%CI 1.22–4.52], *p = 0.015*) increased the risk of MG infection.

Coinfection with CT was found in 18.7% of MG-infected patients (20 women and 7 men) and coinfection with NG was found in 5.55% of MG-infected patients (4 women and 4 men).

### Risks factors associated with MG infection in multivariate analysis (Table 1)

In multivariate analysis, the risk of MG infection was independently associated with multiple partners (6–10 partners: aPR 2.55 [95%CI 1.01–24.63], *p < 0.048;* > 10 partners: aPR 4.33 [95%CI 1.60–11.73], *p < 0.004*), a previous history of STI (aPR 1.89 [95%CI 1.08–3.32], *p < 0.026*), non-use of condoms (aPR 2.56 [95%CI 1.36–4.80], *p < 0.003*) and co-infection with CT (aPR 2.56 [95%CI 1.18–5.57], *p < 0.001)*. Being born in France Mainland (aPR 1.72 [95%CI 0.95–3.09], *p = 0.070*) and precarity (aPR 1.74 [95%CI 0.96–3.13], *p = 0.065*) were on the verge of statistical significance.

**Table 1:** Risk factors associated with *Mycoplasma genitalium* infection: multivariate analysis using Poisson regression, total samples, Reunion Island (*N* = 1263)

Variables	Adjusted PR [95% CI]	*p-*value
Non-use of condoms	2.56 [1.36–4.80]	0.003
Precarity^a^	1.74 [0.96–3.13]	0.065
Place of birth		
Reunion Island	1	
France Mainland	1.72 [0.95–3.09]	0.073
Number of sexual partners in the past year		
0–1	1	
2	0.91 [0.33–2.46]	0.855
3–5	1.89 [0.82–4.31]	0.130
6–10	2.55 [1.01–6.45]	0.048
> 10	4.33 [1.60–11.73]	0.004
Previous history of STI	1.89 [1.08–3.32]	0.026
Co-infection with *Chlamydia trachomatis*	2.56 [1.18–5.57]	0.017

*STI* sexually transmitted infection, *PR* prevalence ratio, *MG Mycoplasma genitalium*, *CI* confidence interval, ^a^defined as: living without employment and social security benefit

The same risk factors were found in the subpopulation from whom urogenital samples were collected.

## Discussion

This study estimated the prevalence of MG in the population attending an STI clinic in Reunion Island. The overall prevalence of MG was 4.88%, a figure higher than that reported in a similar study conducted in 16 STI clinics of France Mainland between September 2014 and January 2015 (3.40%, *p = 0.013*) [4]. Our finding supports an earlier study which found MG infection to be a frequent STI, notably the second most prevalent in Europe [5].

In our study, the prevalence of MG infection in Reunion Island was slightly higher in women than men regardless of age. However, this difference was not statistically significant. The risk of MG infection increased with age until 30 years, though the difference in prevalence between age groups reached statistical significance only in women. As suggested in other studies [4, 8], the risk of MG infection likely increases with longer exposure to high-risk behaviour in young adults and then decreases with the adoption of healthy sexual behaviour beyond the age of 30.

No patient receiving HIV pre-exposure prophylaxis was infected with MG, contrary to the situation in other countries [9, 10].

To prevent overprescription of azithromycin and the resulting increase in macrolide resistance, the latest UK guideline recommends MG screening only in symptomatic patients [5]. In our study (designed before these guidelines were published), screening was performed in both symptomatic and asymptomatic patients. Accordingly, a higher prevalence of MG infection was found in patients engaging in high-risk sexual behaviour—especially precarious women aged under 25 years, all of whom were asymptomatic. This finding contrasts with the situation in France Mainland, where the overall prevalence of MG is also higher in women but the highest prevalence is observed in MSM aged 35–44 years [4].

The increased risk of MG infection in young precarious women presents two problems for Reunion Island, both of which are made worse in the absence of routine MG screening in STI clinics. Firstly, similar to CT infection, MG infection can cause pelvis inflammatory disease and infertility even in asymptomatic women [3, 11]. It is therefore likely that symptom-based screening alone causes a large number of MG-infected patients to go untreated and to develop severe complications. Secondly, young precarious women in Reunion Island, which is characterised by high rates of poverty and the highest rate of abortion in France, are unlikely to adhere to the 7-day doxycycline treatment recommended by current guidelines for CT-infected patients. In this context, single-dose azithromycin remains the treatment of choice for CT. By contrast, Reunionese patients infected with MG alone should ideally be given azithromycin for 3 days to ensure successful treatment. Consequently, and given the large number of patients co-infected with CT in our study, routine screening of at-risk patients, and especially of women aged under 25 years, should be continued to optimize the duration of treatment with azithromycin [12]. In our opinion, this is the best strategy to reduce the prevalence of MG infection and its related complications, while also preventing the development of macrolide resistance in Reunion Island. Indeed, the French national reference centre reported an average prevalence of MG resistance to azithromycin < 6% in French overseas territories for the year 2018 [13]. Moreover, at the time of writing, only one (unpublished) case of macrolide resistance had been reported in our STI clinic, where patients infected with MG were treated with 5-day azithromycin in 2018. Unfortunately, France Mainland-born patients, who had a high prevalence of MG infection in our study (aPR 1.72 [95%CI 0.95–3.09], *p = 0.07*), may be bringing resistance strains of MG to Reunion Island, the problem being far more acute in Europe at the moment.

## Conclusion

Compared to other countries, the prevalence of MG is high in Reunion Island, especially in women aged under 25 years, and co-infection with CT is common. In view of this, routine MG screening and treatment should be performed in at-risk women and co-infection with MG should be considered when deciding on treatment for CT, particularly in regions where azithromycin is still in use.

## Supplementary Information

**Additional file 1: Appendix 1.** Baseline characteristics of patients examined at Saint-Pierre STI clinic, Reunion Island (*N* = 2467). **Appendix 2.** Prevalence of *Mycoplasma genitalium* by sample type, sex, age, sexual behaviour and social status, Saint-Pierre STI clinic, Reunion Island (*N* = 2069).

## Data Availability

The data used during the study are available from the corresponding author.

## Abbreviations

STI: Sexually transmitted infection
MG: *Mycoplasma genitalium*
CT: *Chlamydia trachomatis*
NG: *Neisseria gonorrhoeae*
CI: Confidence interval
PR: Prevalence ratio
MSM: Men who have sex with men
aPR: Adjusted prevalence ratio
