# Supplementary material for: Prevalence and risk factors of Mycoplasma genitalium infection in patients attending a sexually transmitted infection clinic in Reunion Island: a cross-sectional study (2017–2018)
Source: BMC Infect Dis. 2021 May 26;21:482. doi: 10.1186/s12879-021-06193-6 (PMC8157434; doi:10.1186/s12879-021-06193-6)
Supplement: Supplementary file 1 — Additional file 1: Appendix 1. Baseline characteristics of patients examined at Saint-Pierre STI clinic, Reunion Island (N = 2467). Appendix 2. Prevalence of Mycoplasma genitalium by sample type, sex, age, sexual behaviour and social status, Saint-Pierre STI clinic, Reunion Island (N = 2069). [file 12879_2021_6193_MOESM1_ESM.docx]

**To supporting information:**

**Prevalence and risk factors of *Mycoplasma genitalium* infection in patients attending a sexually transmitted infection clinic in Reunion Island: a cross-sectional study (2017-2018)**

Roxane Begnis^1^, Nicolas Bouscaren^2^, Loic Raffray^3^, Cécile Saint Pastou Terrier^1^, Fanny Andry^1^, Malik Boukerrou^4^, Yatrika Koumar^1^, Marie-Pierre Moiton^5^, Patrick Gerardin^2^ and Antoine Bertolotti^1,2*^

¹ CHU Réunion, Service des Maladies Infectieuses - Dermatologie, Saint Pierre, La Réunion, France

^2^ Inserm CIC1410, CHU Réunion, Saint Pierre, La Réunion, France

3 CHU Réunion, Service de Médecine Interne - Dermatologie, Saint Denis, La Réunion, France

^4^ CHU Réunion, Service de Gynécologie et Obstétrique, Saint-Pierre, La Réunion, France

^5^ CHU Réunion, Service des Maladies Infectieuses, Saint-Denis, La Réunion, France

*Correspondence: antoine_bertolotti@yahoo.fr

**Appendix 1** Baseline characteristics of patients examined at Saint-Pierre STI clinic, Reunion Island (N=2,467)

| **Variables** | **All Patients (n=2069)** | **Men (n=1090)** | **Women (n= 1354)** |
| --- | --- | --- | --- |
| Age (md=54) |  |  |  |
| <18 years | 221 (9.2) | 50 (4.7) | 171 (13.0) |
| 18 to 25 years | 777 (32.2) | 288 (26.9) | 483 (36.6) |
| 25 to 30 years | 486 (20.1) | 197 (18.4) | 283 (36.6) |
| >30 years | 929 (38.5) | 535 (50.0) | 383 (29.0) |
| Previous history of STI (md=886) | 562 (35.6) | 359 (35.8) | 195 (34.9) |
| Prostitution (md=0) | 50 (2.0) |  |  |
| Place of birth (md=0) |  |  |  |
| France Mainland | 504 (20.4) | 324 (29.7) | 178 (13.2) |
| Reunion Island | 1963 (79.6) | 766 (70.3) | 1176 (86.9) |
| Employment (md=0) | 750 (45.7) | 510 (50.0) | 236 (39.4) |
| Precarity* (md=9) | 1,387 (56.4) |  |  |
| Pregnancy$ | 24 (1.8) | NA | 24 (1.8) |
| Men who have sex with men | 277 (25.4) | 277 (25.4) | NA |

STI: sexually transmitted infection; * defined as: living without employment and social security benefits. ^$^ only for women. md= missing data.

**Appendix 2** Prevalence of *Mycoplasma genitalium* by sample type, sex, age, sexual behaviour and social status, Saint-Pierre STI clinic, Reunion Island (N=2,069)

|  | All patients (n=2069) | | | | Urogenital samples (n=1,987) | | | | Anal samples (n=327) | | | |
| --- | --- | --- | --- | --- | --- | --- | --- | --- | --- | --- | --- | --- |
| Variables | % [95% CI] | *p* value*^#^* | PR [95% CI] | *p* value*^##^* | % [95% CI] | *p* value*^#^* | PR [95% CI] | *p* value*^##^* | % [95% CI] | *p* value*^#^* | PR [95% CI] | *p* value*^##^* |
| Women | 5.53 [4.27-7.04] |  |  |  | 5.33 [4.07-6.86] |  |  |  | 2.24 [0.46-6.54] |  |  |  |
| Men | 4.13 [2.89-5.72] |  |  |  | 3.22 [2.12-4.68] |  |  |  | 3.76 [1.51-7.75] |  |  |  |
| Men who have sex with men | 7.56 [4.02-12.92] |  |  |  | 3.33 [1.08-7.78] |  |  |  | 4.72 [1.73-10.28] |  |  |  |
| Men who have sex with women | 3.06 [1.78-4.90] |  |  |  | 3.07 [1.79-4.91] |  |  |  | 0.00 |  |  |  |
| Women age group |  | 0.03 |  | 0.02 |  | 0.04 |  | 0.03 |  | 0.38 |  | 0.24 |
| <18 years | 5.84 [2.67-11.09] |  | 2.21 [0.88-5.56] |  | 6.04 [2.76-11.47] |  | 2.45 [0.94-6.34] |  | 0.00 |  | 1 |  |
| 18-25 years | 6.64 [4.41-9.59] |  | 2.51 [1.1-5.31] |  | 6.42 [4.19-9.41] |  | 2.60 [1.18-5.74] |  | 4.55 [0.55-16.42] |  | 1.88x10^7^ |  |
| 25-30 years | 7.95 [4.79-12.41] |  | 3.00 [1.36-6.64] |  | 7.42 [4.32-11.89] |  | 3.01 [1.30-6.97] |  | 4.55 [0.12-25.33] |  | 1.88x10^7^ |  |
| >30 years | 2.65 [1.21-5.02] |  | 1 |  | 2.47 [1.07-4.87] |  | 1 |  | 0.00 |  | NA |  |
| Men age group |  | 0.5 |  | 0.27 |  | 0.15 |  | 0.07 |  | 0.53 |  | 0.54 |
| <18 years | 0 |  | 0 |  | 0.00 [0.00-9.97] |  | 0.00 |  | 0.00 |  | NA |  |
| 18-25 years | 3.52 [1.52-6.94] |  | 0.79 [0.35-1.81] |  | 1.38 [0.28-4.02] |  | 0.33 [0.10-1.13] |  | 6.67 [1.37-19.48] |  | 3.10 [0.52-18.55] |  |
| 25-30 years | 4.91 [2.12-9.67] |  | 1.10 [0.48-2.51] |  | 4.40 [1.77-9.07] |  | 1.06 [0.44-2.57] |  | 3.03 [0.08-16.88] |  | 1.41 [0.13-15.54] |  |
| >30 years | 4.46 [2.69-6.96] |  | 1 |  | 4.14 [2.41-6.62] |  | 1 |  | 2.15 [0.26-7.77] |  | 1 |  |
| Previous history of STI |  | 0.001 |  | 0.002 |  | 0.009 |  | 0.014 |  | 0.56 |  | 0.56 |
| Yes | 6.75 [4.45-9.82] |  | 2.38 [1.38-4.11] |  | 5.4 [3.33-8.42] |  | 2.16 [1.18-3.97] |  | 3.70 [1.36-8.06 |  | 1.44 [0.41-5.11] |  |
| No/unknowm | 2.83 [1.83-4.18] |  | 1 |  | 2.52 [1.58-3.81] |  | 1 |  | 2.56 [0.70-6.57] |  | 1 |  |
| Prostitution |  | 0.044 |  | 0.059 |  | 0.164 |  | 0.184 |  | 0.57 |  | 0.45 |
| Yes | 12.50 [3.41-32.00] |  | 2.70 [0.97-7.14] |  | 9.38 [1.93-27.40] |  | 2.18 [0.69-6.9] |  | 0.00 |  | NA |  |
| No/unknowm | 4.76 [3.86-5.81] |  | 1 |  | 4.30 [3.43-5.32] |  | 1 |  | 3.15 [1.51-5.80] |  | 1 |  |
| Precarity* |  | 0.007 |  | 0.015 |  | 0.28 |  | 0.044 |  | 0.71 |  | 0.73 |
| Yes | 6.76 [3.70-11.35] |  | 2.35 [1.22-4.52] |  | 5.42 [2.71-9.70] |  | 2.19 [1.06-4.55] |  | 4.23 [0.87-12.35] |  | 1.29 [0.32-5.16] |  |
| No | 2.88 [1.86-4.25] |  | 1 |  | 2.47 [1.53-3.78] |  | 1 |  | 3.28 [1.20-7.14] |  | 1 |  |
| Use of condoms |  | 0.04 |  | 0.057 |  | 0.039 |  | 0.06 |  | 0.095 |  | 0.1509 |
| Yes | 6.50 [3.46-11.12] |  | 1 |  | 5.73 [2.86-10.25] |  | 1 |  | 7.89 [1.63-23.07] |  | 0.34 [0.09-1.31] |  |
| No | 3.40 [0.02-4.67] |  | 0.52 [0.28-0.98] |  | 2.84 [1.91-4.05] |  | 0.50 [0.25-0.99] |  | 2.68 [1.08-5.53] |  | 1 |  |
| Number of sexual partners in the past year |  | <.001 |  | 0.003 |  | <.001 |  | 0.023 |  | 0.145 |  | NA |
| 0-1 | 2.11 [0.96-4.01] |  | 1 |  | 2.22 [1.02-4.22] |  | 1 |  | NA |  | 1 |  |
| 2 | 2.39 [1.03-4.70] |  | 1.13 [0.43-2.92] |  | 1.87 [0.68-4.06] |  | 0.84 [0.30-2.36] |  | 0.33 [0.39-1.18] |  | NA |  |
| 3-5 | 4.49 [2.70-7.01] |  | 2.13 [0.96-4.70] |  | 3.45 [1.89-5.80] |  | 1.55 [0.67-3.59] |  | 4.16 [1.13-1.06] |  | NA |  |
| 6-10 | 6.21 [2.98-11.42] |  | 2.94 [1.19-7.24] |  | 6.67 [3.20-12.26] |  | 3.00 [1.22-7.38] |  | 1.79 [0.05-9.95] |  | NA |  |
| >10 | 12.50 [5.40-24.63] |  | 5.92 [2.28-15.34] |  | 8.47 [2.75-19.78] |  | 3.81 [1.28-11.38] |  | 8.82 [1.82-25.79] |  | NA |  |

STI: sexually transmitted infection, PR: prevalence ratio; CI: confidence interval; NA: not applicable; * defined as: living without employment and social security benefits; ^#^Chi^2^ or Fisher test; ^##^Poisson regression
